# Risk Factors of Health-Related Quality of Life among Gastrointestinal Cancer Survivors in the U.S.: With a Focus on Social and Behavioral Determinants of Health (SBDH)

**DOI:** 10.3390/ijerph20176676

**Published:** 2023-08-29

**Authors:** Claire J. Han, Fode Tounkara, Matthew Kalady, Anne M. Noonan, Natasha R. Burse, Electra D. Paskett, Diane Von Ah

**Affiliations:** 1 Center for Healthy Aging, Self-Management and Complex Care, College of Nursing, Ohio State University, Columbus, OH 43210, USA; vonah.1@osu.edu; 2 Cancer Treatment and Research Center, Ohio State University-James, Columbus, OH 43210, USA; fode.tounkara@osumc.edu (F.T.); matthew.kalady@osumc.edu (M.K.); anne.noonan@osumc.edu (A.M.N.); electra.paskett@osumc.edu (E.D.P.); 3 Department of Biomedical Informatics, College of Medicine Ohio State University, Columbus, OH 43210, USA; 4 Division of Colon and Rectal Surgery, Ohio State University-James, Columbus, OH 43210, USA; 5 GI Medical Oncology Selection, Ohio State University-James, Columbus, OH 43210, USA; 6 School of Nursing, University of North Carolina, Chapel Hill, NC 27514, USA; naburse@unc.edu; 7 Division of Epidemiology, College of Public Health, Ohio State University, Columbus, OH 43210, USA

**Keywords:** gastrointestinal, cancer survivor, social and behavioral determinants of health, health-related quality of life

## Abstract

Background: Increasing numbers of long-term gastrointestinal (GI) cancer survivors highlight the importance of understanding the factors contributing to their health-related quality of life (HRQoL). We investigated the risk factors of HRQoL, including demographics, clinical characteristics, and social and behavioral determinants of health (SBDH). Methods: Data on adult GI cancer survivors (*n* = 3201) from the Behavioral Risk Factors Surveillance System (BRFSS) surveys from 2014–2021 (except for 2015) were analyzed. Unadjusted/adjusted logistic regression was used. Results: The majority were women (54%) and white (78%), with a median age of 67. Survivors who were 65 years or older, diagnosed with colorectal cancer, or who had fewer comorbidities were more likely to report significantly better HRQoL. Significant social factors of poor HRQoL included unmarried, racial and ethnic minorities, poor socioeconomic status, and poor healthcare access. Significant behavioral factors of poor HRQoL were lack of physical activity, heavy alcohol consumption, and current smoking, with lack of physical activity being the most significant factor. Conclusions: The SBDH has a critical role in HRQoL. Future studies are warranted to develop a tailored survivorship intervention, such as physical rehabilitation, and to explore machine learning/artificial intelligence-based predictive models to identify cancer survivors at a high risk of developing poor HRQoL.

## 1. Introduction

Gastrointestinal (GI) cancer is a group of cancers that can affect any part of the GI tract, such as esophageal, gastric, colorectal, anal, gall bladder, pancreatic, or liver [1]. This type of cancer is among the leading causes of death in the United States (U.S.) [2], estimated to account for 34% of cancer incidence [1]. The 5-year overall age-standardized relative GI cancer survival rate is rising due to improvements in early identification and treatment in the U.S. in all combined cancer stages and GI cancer types (from 42% in 1975–1990 to 94% in 2012–2018) [2]. It is predicted that by 2050, there will be 350,000 GI cancer survivors living in the U.S. [1,2,3].

As more GI cancer survivors live longer, their health-related quality of life (HRQoL) [3] becomes increasingly significant. Many GI cancer survivors experience poor HRQoL [4,5]. Indeed, a growing number of GI cancer survivors are not only living longer but are also burdened with the risk of cancer recurrence, financial distress, and the long-term consequences of cancer and its symptoms [5,6]. The multiple burdens for GI cancer survivors can significantly impact their HRQoL [2,4,5,7]. 

Notably, significant cancer survivorship disparities were observed across various social and behavioral determinants of health (SBDH), such as race, income status, education levels, and health risk behaviors [8,9]. Therefore, understanding the associations of SBDH factors with the HRQoL of GI cancer survivors can inform targeted interventions to improve their overall well-being. However, identifying GI cancer survivors at a high risk of developing poor HRQoL is under-investigated [10]. 

To our knowledge, while numerous studies have shown that SBDH has a significant impact on cancer survival and mortality rates [9,11,12], very few studies have examined these SBDH factors in relation to the HRQoL of cancer survivors in the U.S. [13]. For example, higher income was associated with better HRQoL, whereas lower educational status negatively impacted HRQoL among Hispanic/Latino-American cancer survivors in mixed cancer types [14] or in breast cancer survivors [15,16]. Burse et al. [13] also examined the association between SBDH and HRQoL in cancer survivors with mixed cancer types in the U.S. The study found that current smoking was positively and significantly associated with poor physical HRQoL, but healthy eating (i.e., fruit and vegetable consumption), heavy alcohol consumption, and health care coverage were not associated with HRQoL after covariate adjustment. However, these studies [13,14,15,16] were limited in identifying the most significant SBDH risk factors of poor HRQoL, specific to GI cancer survivors. 

Understanding the roles of SBDH on HRQoL among GI cancer survivors is crucial to gaining insight into the specific social challenges and needs of this population. Lower socioeconomic status and poor lifestyle, including health risk behaviors, are associated with a higher risk of GI cancer, as well as higher mortality and recurrence rates in patients with GI cancers [15]. Poor diet (e.g., red meat, heavy alcohol, or fast food consumption), sedentary lifestyles, and smoking status contribute to GI cancer development as well as poor disease prognosis [17]. Overall, SBDH may play a role not only in the risk of GI cancer development but also in hastening the onset of symptoms and poor HRQoL. Researchers have identified that poor SBDH was associated with severe and frequent GI and psychological symptoms [4,5], which contribute to the risk of poor physical and mental HRQoL among GI cancer survivors. Marco et al. [18] reported that cancer survivors with prostate, melanoma, gynecological, or urological cancers had higher HRQoL scores than those with colorectal cancer. Thus, HRQoL can differ by cancer type, meaning it is important to identify SBDH risk factors of HRQoL specific to GI cancer survivors instead of examining these aspects in relation to all combined cancer types [18]. 

Therefore, the purpose of this study is to examine the associations of SBDH with HRQoL among GI cancer survivors in the U.S. Our aims are: (1) to identify the most influential or significant risk factors of poor general, physical, and mental HRQoL outcomes, including demographic and clinical characteristics, and SBDH (e.g., race, health risk behaviors, income, education, healthcare access, home ownership); and (2) to quantify the associations of SBDH with HRQoL following covariate adjustment among GI cancer survivors. This study focuses on SBDH as the primary risk factor for poor HRQoL. The significant demographic and clinical characteristics related to HRQoL that were initially identified (Aim 1) were subsequently adjusted as covariates in Aim 2.

## 2. Materials and Methods

### 2.1. Data Source and Study Population

A nationwide telephone survey known as the Behavioral Risk Factors Surveillance System (BRFSS) was launched by the Centers for Disease Control and Prevention (CDC) in 1984 [19]. In all 50 U.S. states, the District of Columbia, and three U.S. territories, BRFSS interviewers gather information on health-related behaviors, sociodemographic factors, the top preventable causes of death, and preventive health practices among non-institutionalized residents (i.e., residents 18 years of age or older). The BRFSS conducts surveys over landlines or cellular telephones using a random digit dialing sampling technique. The validity and reliability of BRFSS data have been demonstrated [19]. A secondary data analysis was conducted using publicly available BRFSS survey data. The Institutional Review Board (IRB) waived approval for this study.

A cross-sectional study was conducted by combining BRFSS data pertaining to GI cancer survivors from 2014 to 2021, with 2015 excluded due to a lack of availability of relevant data. Survey questions about diet were asked only in the survey for the years 2017, 2019, and 2021. Surveys from these specific years were merged to examine diet as a risk factor for HRQoL. Individuals ≥ 18 years of age who self-reported a personal history of esophageal, stomach, colon, rectal, liver, or pancreatic cancers were included as adult GI cancer survivors in this study. Individuals were excluded if they refused to respond to any of the survey questions or had missing responses or values for any of the included variables used in this study.

### 2.2. Measures

#### 2.2.1. Primary Outcomes of Interest

The CDC HRQoL-4 measure was used in this study. This measure includes self-reported general, physical, and mental health status and usual activity limitations according to physical or mental health status [14]. The primary outcomes included all three items of the HRQoL-4 measure—general, physical, and mental health. The following survey questions were used to measure each health status [19]: For general health, “Would you say that in general, your health is excellent, very good, good, fair or poor?”; for physical health, “Now thinking about your physical health, which includes physical illness and injury, for how many days during the past 30 days was your physical health not good?”; and for mental health, “Now thinking about your mental health, which includes stress, depression, and problems with emotions, for how many days during the past 30 days was your mental health not good?” The cutoff for categorizing the primary outcomes was validated by the CDC [20]. General health was dichotomized as “better” if answered as “excellent”, “very good”, or “good” versus “poor” if answered as “fair” or “poor”. Physical and mental health status were also dichotomized as “better” versus “poor”. Better physical health was defined as having 0–13 physically unhealthy days, while poor physical health was defined as having 14 or more such days. Similarly, better mental health was defined as having 0–13 mentally unhealthy days, while poor mental health was defined as having 14 or more such days. 

#### 2.2.2. Demographic and Clinical Characteristics

In these BRFSS data, we included age, sex, GI cancer types, and comorbidities as demographic and clinical characteristics were included to examine associations with HRQoL as potential covariates.

#### 2.2.3. Social and Behavioral Determinants of Health (SBDH)

**Figure 1:**
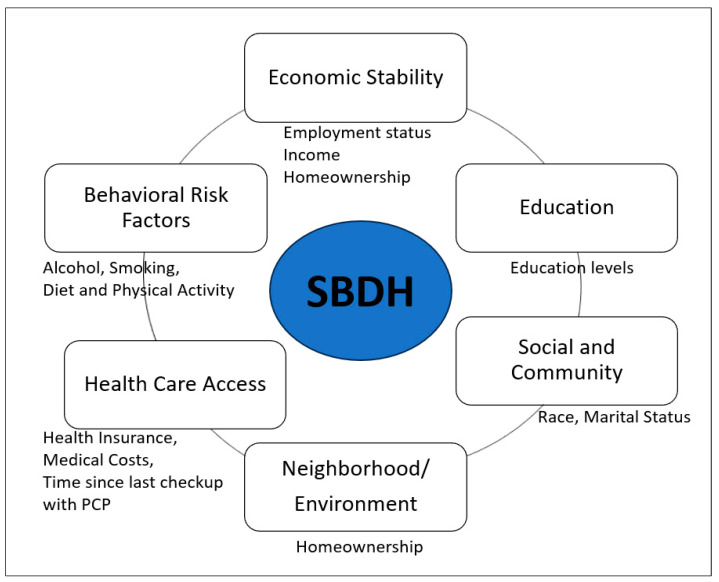
Healthy People 2030 framework’s five domains of social determinants of health and behavioral risk factors are relevant to the social and behavioral determinants of health (SBDH) in the current study.

In this study, SBDH was measured as a risk factor for poor HRQoL, including social determinants of health (SDOH) and health risk behaviors. The national health initiative, Healthy People 2030 [21], organizes SDOH into five key areas: economic status, education, social and community context, healthcare access and quality, and neighborhood and built environment. To correspond BRFSS data to the SDOH in accordance with Healthy People 2030, social community context (e.g., race, ethnicity, marital status), education, economic status (e.g., annual household income, employment status, homeownership—rent versus own home), and healthcare access (e.g., health care insurance coverage, time since the last health checkup, concerns of medical costs limited the number of doctor visits) were included. There were no available variables of BRFSS data that matched up with the neighborhood and built environment area of the Healthy People 2030. This study further included behavioral risk factors, including diet, physical activity, alcohol consumption, and smoking status (Figure 1). The diet variable (e.g., fruit and vegetable consumption per day) was grouped into two categories: “less than one time per day” and “one or more times per day”. The BRFSS physical activity questions, “Adults who reported doing overall routine physical activity or exercise during the past 30 days other than their regular jobs?” and “No physical activity or exercise during the past 30 days”, were used for the current study. The BRFSS defined heavy drinking as having more than seven drinks per week for women and more than 14 drinks per week for men. Current smoking was considered a binary variable (either “yes” or “no”) [22]. None of the variables related to social and community context, quality of care, or environmental factors (e.g., zip code, poverty index, environmental safety, transportation) were available in the BRFSS dataset of GI cancer survivors. 

### 2.3. Statistical Analysis

The BRFSS was designed to obtain health-related information on the population of interest (i.e., the adult U.S. population residing in different states) [21]. Data weighting helped make these sample data more representative of the population from which these data were collected. The BRFSS data weights incorporated both population characteristics and BRFSS survey design, while the weighting methodology consisted of (1) weight or factors design and (2) a method for adjusting the population’s demographics, such as ranking or interactive proportional fitting [23]. Complex survey procedures with appropriate stratification and weighting of these data were applied to this study’s sample. 

The statistical analysis for this study involved a combination of descriptive statistics, univariate analysis, and multivariate logistic regressions. All of our variables were normally distributed with skewness and kurtoses within the range of ±2, indicating the normal distribution; thus, we used parametric statistical methods. To examine the univariate correlations with HRQoL outcomes, the Chi-square test was used to analyze categorical independent variables, and the Analysis of Variance (ANOVA) was used for continuous independent variables. Logistic regression was then used to estimate the odds ratios (ORs) and 95% confidence intervals (CIs) for the association between SBDH and each HRQoL outcome. The SBDH factors were only included in the regression models if they were significantly associated with HRQoL outcomes. Most of the independent variables in our regression models were categorical variables. Given the assumption that categorical independent variables cannot be colinear in the regression model, stepwise eliminations were performed using multivariate regression models to select a parsimonious model while minimizing the collinearity among the variables [24]. The demographic and clinical characteristics significantly associated with HRQoL were adjusted as covariates for multivariate regression models. We also controlled surgery years for all regression models in this study. Univariate (i.e., unadjusted, other predictors) ORs and adjusted OR from multivariate regression models (AOR, i.e., adjusted other predictors) and 95% CIs were reported for the final models. Cox and Snell R squared and Nagelkerke R squared were used for the model fit test (goodness of fit) to see the variability of the independent variables in explaining the variability of the outcome variables (better versus worse HRQoL measures). All statistical analyses were performed using the R statistical software program. The level of significance for all analyses was set at *p* < 0.05 (two-sided).

## 3. Results

### 3.1. Demographic and Clinical Characteristics 

The unweighted population consisted of 3201 GI cancer survivors. Following population weighting to the respective states, 229,428 adult GI cancer survivors were represented in the combined dataset from 2014 to 2021 (except for 2015). The demographic and clinical characteristics are presented in Table 1. In the main dataset, around half of the GI cancer survivors were 65 years or older (57%), with a median age of 67, and 54% were women (Table 1A). Among the GI cancer survivors, colorectal cancer was the most common cancer (72.5%), followed by liver cancer (10%) and stomach cancer (7.2%). In terms of comorbidities, diabetes (51.3%) was the most common chronic condition among the survivors, followed by chronic arthritis (48.9%). In the subset of the main dataset combining the 2017, 2019, and 2021 surveys with available diet variables (Table 1B), similar results were found: the majority of GI cancer survivors were 65 years or older (68%), women (52%), and had been diagnosed with colorectal cancer (77.1%). Diabetes (54.9%) and chronic arthritis (45.6%) remained the most common chronic conditions. 

### 3.2. HRQoL Outcomes and SBDH 

The HRQoL and SBDH factors are described in Table 2. In the main BRFSS dataset, over half of the GI cancer survivors reported better general (62%) and mental (56%) HRQoL, while 43% reported better physical HRQoL (Table 2A). In the main dataset, half of the GI cancer survivors were married or partnered (50%) and had at least a college education (50%), while 78% were non-Hispanic White. Approximately 57.3% of the cancer survivors had an annual household income of at least $35,000, and 93% had healthcare coverage. Most of the cancer survivors were on retirement benefits (55.3%), homeowners (79%), not heavy drinkers (91%), and not current smokers (83%). Approximately 65% of the GI cancer survivors reported partaking in routine physical activity or exercise over the last month. Similar results were found in the subset of BRFSS data combining the 2017, 2019, and 2021 surveys. In this subset (Table 2B), the majority of participants had consumed fruits (68%) and vegetables (80%) one or more times per day.

### 3.3. Univariate Associations of Demographic and Clinical Characteristics with HRQOL

This study examined specific demographic and clinical characteristics alongside HRQoL to identify potential covariates for primary analyses (i.e., SBDH and HRQoL) (Table 3). Being in an older age group, being married/partnered, and having no diagnosis of asthma were significantly associated with better general and mental HRQoL (all *p* < 0.001). Several chronic conditions were associated with poor HRQoL in all three HRQoL outcomes. Having ever been diagnosed with colorectal cancer—compared with other types of GI cancer such as liver or pancreatic cancers—and having no past medical history of coronary heart disease or chronic kidney disease were significantly associated with better general and physical HRQoL (all *p* < 0.05).

### 3.4. Univariate Associations of SBDH with HRQoL

The univariate associations of SBDH with HRQoL are described in Table 4. Many SBDH factors significantly increase the risk of poor general, mental, and physical HRQoL. However, daily fruit consumption, health insurance, and medical costs were not significantly associated with HRQoL measures. 

Given the significant correlation of several SBDH with HRQoL (Table 4), the potential impact of SBDH on HRQoL was further quantified using multivariate regression models (Table 5). The unadjusted and adjusted ORs of each HRQoL outcome in relation to the SBDH are shown in Table 5. In multivariate analyses (AOR) (Table 5), R squared for each HRQoL outcome indicates a moderating fit of the regression model (e.g., the Nagelkerke R squared value of 0.42 in general HRQoL means that the independent variables can explain about 42% of the variation in the general HRQoL outcome, while the remaining 58% is due to other factors. In the adjusted, multivariate logistic models with adjusted covariates (e.g., survey years, age, GI cancer types, comorbidities) (Table 5), among the SDOH, being non-Hispanic Whites (AOR = 1.02, 95% CI: 1.01–1.12), being married/partnered status (AOR = 1.06, 95% CI = 1.01–1.23), having higher education levels (AOR = 1.33, 95% CI = 1.11–1.52), being employed (AOR = 1.12, 95% CI = 1.06–1.13), having higher income (AOR = 1.08, 95% CI = 1.05–1.32), and healthcare access within the past year with a primary care provider (AOR = 1.41, 95% CI = 1.13, 1.77), were associated with better general HRQoL. Similar results were found for mental and physical HRQoL. Regarding the behavioral determinants of health, alcohol consumption, current smoking status, and lack of physical activity were significantly associated with poor general HRQoL. Among all SBDH in this study, physical activity participation was the most significant risk factor for better HRQoL (AOR = 1.98 for general, AOR = 1.74 for mental, and AOR = 1.94 for physical HRQoL), followed by better healthcare access (i.e., frequent health checkup) (AOR = 1.41 for general, AOR = 1.46, for mental, and AOR = 1.49 for physical HRQoL).

## 4. Discussion

This study marks the initial exploration of SBDH risk factors on HRQoL among U.S. adults with various GI cancer types, encompassing an array of SBDH. The findings underscore the significant associations of poor HRQoL with many individual-level demographic and clinical characteristics as well as social-level (i.e., SBDH). Poor status SBDH—low economic stability, poor healthcare access, non-Hispanic Blacks, poor health risk behaviors—were significantly associated with poor general, mental, or physical HRQoL. Lack of physical activity and less healthcare access (i.e., less frequent health checkups) were the major SBDH factors of poor HRQoL in all three HRQoL outcomes.

This study is one of the few that examines HRQoL in relation to different GI cancer types. The analyses demonstrated significant evidence of GI cancer-type differences in general and physical HRQoL outcomes. Notably, GI cancer survivors diagnosed with esophageal, liver, pancreatic, or stomach cancers were more likely to report poor general and physical HRQoL compared with colorectal cancer survivors. One possible reason for this relates to how there is a higher cancer burden in certain GI cancer types compared with colorectal cancer. For example, liver and pancreatic cancers generally have a poor prognosis and are often diagnosed at advanced stages with high mortality rates [9]. Furthermore, this can be due to the better prognosis of colorectal cancer compared with liver or pancreatic cancer, as the early screening and diagnosis of colorectal cancer are well-established [25]. Future consideration of a weighted regression model is suggested to examine the contribution of each GI cancer type on HRQoL. Interestingly, the older adults (i.e., 65 years or older) reported better general and mental, but not physical, HRQoL in this study. This could be due to the fact that older adults have higher resilience and are more capable of managing or resolving conflicts, regardless of socioeconomic status and personal health conditions, or older adults in the retirement stage might have fewer responsibilities in terms of major life events, compared with younger adults (i.e., 18–64 years old) [26]. It is important to identify different risk factors that contribute to HRQoL between younger and older age groups in view of providing age-tailored cancer survivorship interventions. 

In this study, non-Hispanic Black and other racial and ethnic groups had a higher prevalence of reporting poor general HRQoL at 22.7%, compared to 17% who reported better general HRQoL (*p* < 0.001). Similarly, in a Surveillance, Epidemiology, and End Results (SEER) study, non-Hispanic White cancer survivors had better HRQoL scores than Black and Hispanic cancer survivors in the U.S. [27]. This might be due to the structural racism that exists in the U.S., including in the healthcare system, which could result in poor healthcare access, low health literacy, disparities in cancer survivorship care and treatment options, comorbidity burdens, socioeconomic status, insurance coverage, and lack of community resources and policy support [27]. Marital status, which is often used as a proxy of social support, was significantly related to better general and mental HRQoL [27]. Consistent with previous research [13,27], being married/partnered, having high-income status, high educational levels, owning a home, frequent healthcare access, and optimal health behaviors were associated with better HRQoL outcomes. Marital status, which is often used as a proxy of social support, was significantly related to better general and mental HRQoL [28]. Future studies should explore the influence of structural racism and social support on HRQoL outcomes [29]. 

In analyzing the impact of SBDH on HRQoL after covariate adjustment, this study showed that current engagement in physical activity was the most impactful factor related to better HRQoL outcomes. Numerous studies have demonstrated significant associations between physical activity and better mental and physical HRQoL among cancer survivors [30,31,32,33]. The mechanisms through which physical activity improves the HRQoL are unknown. One explanation could be symptoms such as fatigue and psychological distress acting as a mediator between physical activity and HRQoL in cancer survivors [34,35,36]. Other potential mechanisms that could explain the link between physical activity and HRQoL include systemic inflammation, the release of endorphins, or the blocking or diminishing of external or internal forces that cause stress in cancer survivors [36,37,38]. A significant association between daily fruit and vegetable consumption and HRQoL in the adjusted models was not found. It is possible that daily fruit and vegetable consumption may not be a sufficient measure for diet or do not fully reflect the nutritional status, such as diet quality and food groups, which is significantly associated with the risk of GI cancers and cancer survivorship [37]. Other health risk behaviors, including smoking status and alcohol habits, were also associated with HRQoL. These health risk behaviors can increase the risk of cancer recurrence and poor disease progress in cancer survivors in the long term [38]. 

These findings have important implications for clinical practice and public health interventions. They suggest that lifestyle interventions—specifically targeting physical activity—and the screening and prevention of health risk behaviors, as well as the promotion of healthy behaviors, must be an integral part of cancer survivorship care. For example, healthcare providers should emphasize the importance of physical activity and smoking cessation for GI cancer survivors and consider referral to functional or mental health rehabilitation for those with poor mental or physical HRQoL. Policymakers should consider supporting a community-based cancer survivorship program to improve healthcare access. The findings of the study could be leveraged to develop a machine learning and artificial intelligence-based predictive model of HRQoL. These models could incorporate data on demographics, clinical characteristics, and SBDH risk factors to identify patients at risk of poor HRQoL and create personalized interventions to improve their HRQoL. Furthermore, these models could help identify factors most strongly associated with HRQoL, which could inform the development of more effective interventions.

The strengths of this study include the utilization of BRFSS data as a reliable data source, with national representation to increase the generalizability of the research findings [23]. This study adds to the body of literature by identifying possible risk factors of poor HRQoL with a primary focus on the roles of SBDH, specific to GI cancer survivors. However, this study also involves a number of limitations. First, the study is limited by the cross-sectional nature of these data, which hindered the ability to establish causality. For example, whether poor SDOH status mediates the relationships between racial and ethnic minorities and poor HRQoL could not be determined. Second, self-reported HRQoL data may be subject to reporting bias. Specific symptoms—particularly fatigue, psychological distress, and GI symptoms—as well as dietary factors (e.g., red meat, fruit, and vegetable consumption) have been found to be associated with the HRQoL among GI cancer survivors. However, these variables were not examined in the current study. Additionally, other SBDH factors such as poverty level, neighborhood and environmental factors, and social support level data were not available for this study. Finally, the study could not adjust for cancer stages or types of cancer treatments (e.g., radiation, chemotherapy, surgery) in association with HRQoL in GI cancer survivors [18]. 

## 5. Conclusions

Our findings provide valuable insights into the SBDH and demographic and clinical characteristics that may influence the HRQoL among GI cancer survivors in the U.S. The results of this study highlight the important impact of age, comorbidities, and type of GI cancer (i.e., non-colorectal cancer) on HRQoL. In addition, among multiple SBDH factors, specifically low economic stability, unemployment, poor health care access, smoking/alcohol health risk behaviors, and lack of physical activity, significantly contribute to poor HRQoL among GI cancer survivors. Thus, future studies should consider the comprehensive assessment of HRQoL in view of developing and testing tailored cancer survivorship interventions for GI cancer survivors, particularly those in underserved and racial and ethnic minority populations with social or economic disadvantages. 

## Figures and Tables

**Table 1:** Demographic and Clinical Characteristics among GI Cancer Survivors.

A. Main Dataset (2014 to 2021, Except for 2015) Total Weighted Study *N* = 229,428 *Unweighted n = 3201*			B. Subset of Main Dataset (2017, 2019, and 2021 with Diet Variables) Total Weighted Study *N* = 123,261 *Unweighted n = 835*	
Year, *n* (%)		229,428	Years, *n* (%)	123,261
2014		12,576 (5.5)	2014	not included
2016		20,718 (9.0)	2016	not included
2017		16,870 (7.3)	2017	57,933 (47)
2018		14,128 (6.1)	2018	not included
2019		196 (<0.1)	2019	3,697 (3.0)
2020		141,212 (62)	2020	not included
2021		23,729 (10)	2021	61,631 (50)
**Demographics (% otherwise specified)**				
Age, median (Interquartile range, IQR)		67 (58, 76)	67 (58, 77)	
Age group	18–64	43%	32%	
	65 or older	57%	68%	
Sex	Male	46%	48%	
	Female	54%	52%	
**Clinical Characteristics, *n* (%)**				
Types of GI Cancer	Colorectal (intestine)	72.5%	77.1%	
	Esophageal	5.2%	6%	
	Liver	10%	7.4%	
	Pancreatic	5.1%	4.6%	
	Stomach	7.2%	4.9%	
**Comorbidities, *n* (%)**				
The participants self-reported if they had ever been told by a health professional that they had(“yes”/no”)				
Heart Attack (Yes)		12%	9.2%	
Coronary Heart Disease (Yes)		12%	11%	
Asthma (Yes)		17%	14%	
Stroke (Yes)		8.3%	8.3%	
Chronic Obstructive Pulmonary Disease (Yes)		14.5%	13.2%	
Diabetes (Yes)		51.3%	54.9%	
Chronic Arthritis (Yes)		48.9%	45.6%	
Chronic Kidney Disease (Yes)		8.4%	11%	
Overweight or obese per Body Mass Index (Yes)		29.5%	14%	

Note. Interquartile range, IQR.

**Table 2:** HRQoL Outcomes and SBDH among GI Cancer Survivors (%).

A. Main Dataset (2014 to 2021, Except for 2015) Total Weighted Study *N* = 229,428 *Unweighted n = 3201*			B. Subset of Main Dataset (2017, 2019, and 2021 with Diet Variables). Total Weighted Study *N* = 123,261 *Unweighted n = 835*
**HRQOL OUTCOMES**			
General Health	Poor (38%), Better (62%)		Poor (37%), Better (63%)
Mental Health	Poor (44%), Better (56%)		Poor (13%), Better (87%)
Physical Health	Poor (57%), Better (43%)		Poor (24%), Better (76%)
**SOCIAL AND COMMUNITY CONTEXT**			
Race/Ethnicity	Non-Hispanic White	78%	82%
	Non-Hispanic Black	12%	6%
	Non-Hispanic Other	5.5%	7.8%
	Hispanic	4.5%	4.2%
Marital status	Married/Partnered	50%	51%
	Divorced/Widowed/Single	50%	49%
**EDUCATION**			
High school or less		50%	35%
Attended College or technical school		31%	32%
Graduated from College or technical school		19%	33%
**ECONOMIC STABILITY**			
Employment Status	Employed	28%	25.5%
	Unemployed	11.7%	10.5%
	Retirement Benefits	55.3%	58.5%
	Homemaker	5%	5.5%
Household Income (annual)			
Less than $15,000		9.4%	9.2%
$15,000 to < $25,000		18%	15.4%
$25,000 to < $35,000		15.3%	15.4%
$35,000 to < $50,000		22.7%	22.9%
$50,000 to < $100,000		33.1%	32.2%
$100,000 or more		1.5%	4.9%
Homeownership	Own	79%	79%
	Rent	18%	18%
	Other arrangement	3%	3%
**HEALTHCARE ACCESS**			
Health Insurance	Yes	93%	96.2%
	No	6.2%	3.1%
	Don’t know/Not sure	0.8%	0.7%
Medical Costs: In the past year, could not see doctor due to medical costs?			
(Yes or No)		Yes (8%)	Yes (6.9%)
Health Care Access: Time since last checkup with primary care providers			
Within past years		87%	86%
1 but <2 years ago		7.8%	8%
2 but <5 years ago		3.3%	3%
5 or more years ago		1.6%	2%
Never		0.1%	1%
**BEHAVIORAL RISK FACTORS**			
At least one alcohol in the past 30 days	Yes/No	40%/60%	39%/61%
Heavy drinking per week	Yes/No	9%/91%	7%/93%
Smoking Status	Current	17%	11.5%
	Former	40%	37.5%
	Never	43%	51%
Physical Activity	Yes/No	65%/35%	65%/35%
Diet (Fruits)	Less than one time per day	no data available	29%
	One or more times per day		71%
Diet (Vegetables)	Less than one time per day	no data available	17%
	One or more times per day		83%

**Table 3:** Univariate Associations of Demographic and Clinical Characteristics with HRQOL. (To identify potential covariates between SBDH and HRQOL).

Main Dataset (2014 to 2021, Except for 2015), Total Weighted Study *N* = 229,428, *Unweighted n = 3201*										
Variables		General HRQoL ^a^			Mental HRQoL ^a^			Physical HRQoL ^a^		
		Poor	Better	*Chi^2^*/*p* ^b^	Poor	Better	*Chi^2^*/*p* ^b^	Poor	Better	*Chi^2^*/*p* ^b^
**DEMOGRAPHICS (% OTHERWISE SPECIFIED)**										
Age, Median (IQR)		64 (13)	65 (14)	3.41 0.367 ^c^	57 (15)	60 (15)	2.93 0.314 ^c^	63 (13)	61 (16)	3.01 0.840 ^c^
Age group	18–64	37.5	29.0	15.42	56.5	42.6	16.51	40.5	37.3	3.62
	65 or older	61.5	70.0	**<0.001**	42.5	57.0	**<0.001**	58.8	61.9	440
Sex	Male	46.7	46.0	2.41	38.8	39.5	3.21	45.5	42.3	2.31
	Female	53.3	54.0	0.674	61.2	60.5	0.822	54.5	57.7	0.237
**CLINICAL CHARACTERISTICS** **(%)**										
Types of GI cancer				9.21 **<0.001**			1.21 0.710			12.31 **<0.001**
Colorectal		71.0	83.8		71.9	73.1		68.0	78.3	
Esophageal		6.0	4.2		6.5	5.5		7.3	4.4	
Liver		10.2	4.5		10.0	8.4		10.6	5.8	
Pancreatic		6.3	3.9		4.5	6.6		7.3	5.3	
Stomach		6.6	4.6		7.2	7.5		6.8	6.2	
Comorbidities										
Heart Attack	Yes	18.7	7.5	13.41	16.4	10.4	15.86	18.8	12.2	2.50
	No	80.2	91.9	**<0.001**	82.3	88.2	**0.023**	80.4	86.7	0.003
Coronary Heart Disease				15.42 **<0.001**			1.31 0.380			16.99 **<0.001**
	Yes	18.8	8.0		15.7	12.2		20.3	11.5	
	No	79.3	90.4		82.8	86.0		78.4	86.5	
Asthma	Yes	20.2	11.3	10.51	25.6	15.5	9.48	20.5	18.8	1.21
	No	79.5	88.4	**<0.001**	74.4	84.3	**<0.001**	79.3	80.7	0.576
Stroke	Yes	13.3	5.2	23.22	14.2	8.0	24.48	11.3	8.4	26.51
	No	85.9	94.6	**<0.001**	84.1	91.6	**0.001**	87.9	91.2	**0.002**
COPD	Yes	23.5	9.0	12.22	26.4	16.4	13.56	24.9	15.7	11.24
	No	75.4	90.6	**<0.001**	71.9	61.0	**0.001**	74.1	83.5	**<0.001**
Diabetes	Yes	33.3	21.4	36.21	31.3	25.2	34.59	31.6	26.9	38.83
	No	63.4	76.8	**<0.001**	65.4	71.8	**0.043**	65.4	70.5	**0.023**
Chronic Arthritis	Yes	59.2	42.9	26.08	66.4	51.4	25.57	62.2	54.3	28.21
	No	40.5	56.5	**<0.001**	33.7	48.1	**<0.001**	37.4	45.2	**0.011**
Chronic Kidney Disease										
	Yes	13.0	5.6	15.64	11.7	10.1	1.45	13.8	8.4	16.77
	No	86.0	94.0	**<0.001**	87.1	88.9	0.556	85.2	90.9	**0.012**
Overweight/Obese				1.22 0.886			1.09 0.123			1.29 0.323
	Yes	31.5	31.8		30.6	36.4		31.9	32.9	
	No	64.3	64.3		66.4	59.9		64.5	62.1	

Note. Interquartile range, IQR; Significant findings (*p* < 0.05) were highlighted in bold. ^a^ Levene’s test statistics for the age median variable showed the homogeneity of variance in the ANOVA models: For general HRQoL (0.121, significance = 0.728), for mental HRQoL (0.721, significance = 0.428), and for physical HRQOL (0.851, significance = 0.356). ^b^ Chi-square statistics and *p* values except for the age median variable. ^c^ For the age median variable, F/p statistics from ANOVA were used.

**Table 4:** Univariate Associations of SBDH with HRQoL.

Main Dataset (2014 to 2021, Except for 2015). Total Weighted Study *N* = 229,428, *Unweighted n = 3201*										
SBDH Risk Factors		General HRQoL			Mental HRQoL			Physical HRQoL		
		Poor	Better	*Chi^2^*/*p* ^a^	Poor	Better	*Chi^2^*/*p* ^a^	Poor	Better	*Chi^2^*/*p* ^a^
**SOCIAL AND COMMUNITY CONTEXT (%)**										
Race/Ethnicity:				34.94 **<0.001**			2.26 0.230	80.6	78.3	2.35 0.398
Non-Hispanic White		77.3	83.0		77.4	79.6		6.8	8.9	
Non-Hispanic Black		8.9	6.2		6.2	7.1		3.5	4.1	
Non-Hispanic Other		6.7	4.6		6.2	8.4		3.9	4.0	
Hispanic		4.4	2.4		4.0	3.3		3.4	2.9	
Marital Status:				9.42 **<0.001**			10.32 **0.002**			3.12 0.096
Married/Partnered		44.0	52.2		41.0	49.7		49.3	46.3	
Divorced/Widowed/Single		56.0	47.8		59.0	50.3		50.7	53.7	
EDUCATION (%)										
High school or less		47.2	35.8	21.41 **<0.001**	41.3	38.4	22.52 **0.014**	12.5	9.9	25.62 **<0.001**
Attended College or technical school		30.9	29.4		33.4	32.4		33.1	28.7	
Bachelor’s degree or graduate or more		21.6	34.5		24.9	67.5		54.5	60.9	
**ECONOMIC STABILITY (%)**										
Employment Status:				30.42 **<0.001**			22.31 **<0.001**			29.62 **<0.001**
Employed		17.0	29.0		17.4	27.3		16.0	25.3	
Unemployed		20.1	17.1		45.3	33.0		32.5	18.5	
Retirement Benefits		58.9	48.9		31.1	47.0		45.7	51.7	
Homemaker		4.0	5.0		6.2	2.7		5.8	4.5	
Household Income/year:				36.52 **<0.001**			33.52 **<0.001**			36.31 **0.002**
Less than $25,000		36.4	20.7		42.8	27.1		38.7	29.3	
$25,000 to <$35,000		12.0	10.9		8.7	10.4		10.6	8.9	
$35,000 to <$50,000		12.6	12.8		11.7	15.1		12.4	12.6	
$50,000 to <$100,000		21.9	35.2		22.6	29.1		23.1	30.8	
$100,000 or more		0.8	2.2		0.2	2.8		0.7	1.8	
Homeownership	Own	72.7	81.8	25.1	66.9	75.2	29.3	71.2	75.9	2.31
	Rent	22.9	15.1	**<0.001**	26.1	21.9	**0.012**	24.0	19.9	0.234
**HEALTHCARE ACCESS (%)**										
Health Insurance	Yes	94.2	94.7	1.11	91.0	93.3	1.43	93.9	94.2	2.12
	No	5.1	4.3	0.580	7.5	6.3	0.289	5.3	5.3	0.786
Medical Costs:				1.03 0.101			1.33 0.108			5.21 0.050
In the past year, could not see a doctor due to medical costs. (Yes/No)		Yes (10.3)	Yes (6.6)		Yes (15.1)	Yes (8.9)		Yes (11.9)	Yes (8.1)	
Time since last checkup with PCP:				14.24 **0.021**			10.582 **0.049**			11.52 **0.032**
Within the past year		84.6	87.5		85.4	80.8		84.3	88.3	
More than 1 year ago or never		15.4	12.5		14.6	19.2		15.7	11.7	
**BEHAVIORAL RISK FACTORS (%)**										
At least one alcoholic drink in last month	Yes	28.1	42.3	14.31	32.6	39.9	5.62	28.5	38.1	15.41
	No	71.3	57.0	<**0.001**	67.4	59.6	0.050	70.8	61.4	**0.001**
Heavy drinking per week	Yes	94.7	92.3	15.41 **0.022**	92.8	92.9	1.09 0.184	94.2	93.7	2.31 0.731
	No	4.4	6.7		7.0	5.8		4.9	5.7	
Smoking Status	Current	17.9	8.8	17.63 **<0.001**	28.0	13.3	19.31 **<0.001**	18.8	13.6	23.41 **0.004**
	Former	41.9	38.8		37.3	36.6		41.0	38.1	
	Never	39.4	51.9		33.8	49.4		39.4	48.0	
Physical Activity	Yes	50.5	73.1	24.31	49.3	65.4	26.51	46.5	65.3	25.61
	No	52.8	26.7	**<0.001**	50.2	34.6	**<0.001**	53.0	34.7	**<0.001**
**Subset of the main dataset (2017, 2019, and 2021 with diet variables)** **Weighted Study *N* = 123,261, *unweighted n = 835***										
Diet (Fruits):				1.41 0.290			1.65 0.574			1.39 0.119
Less than one time/day		69.7	64.9		65.6	59.5		72.0	64.8	
One or more times/day		27.0	31.7		31.8	37.9		26.9	31.5	
Diet (Vegetables):				19.51 **0.004**			20.41 **0.025**			18.51 **0.046**
Less than one time/day		80.5	74.4		84.4	71.6		82.8	72.7	
One or more times/ day		15.2	23.3		12.3	25.0		15.6	23.6	

Note. Significant findings (*p* < 0.05) were highlighted in bold. PCP, Primary Care Provider. ^a^ Chi-square statistics and *p* values.

**Table 5:** Potential Impact of SBDH on HRQoL among GI Cancer Survivors.

Exp (B) is an Odds Ratio (OR)/OR as a Univariate Association. AOR as an Adjusted OR That Controls for Other Predictor Variables in Multivariate Regression.	Main Dataset (2014 to 2021, Except for 2015). Total Weighted Study *N* = 229,428, *Unweighted n = 3201*								
	Better General HRQoL (62%, Weighted *n* = 137,905)			Better Mental HRQoL (56%, Weighted *n* = 124,559)			Better Physical HRQoL (43%, Weighted *n* = 98,654)		
	Univariate	Multivariate Model ^b^ Cox and Snell R^2^ (0.37) Nagelkerke R^2^ (0.42)		Univariate	Multivariate Model ^b^ Cox and Snell R^2^ (0.28) Nagelkerke R^2^ (0.35)		Univariate	Multivariate Model ^b^ Cox and Snell R^2^ (0.29) Nagelkerke R^2^ (0.35)	
	OR (95% CI)	AOR (95% CI)	Wald	OR (95% CI)	AOR (95% CI)	Wald	OR (95% CI)	AOR (95% CI)	Wald
**SOCIAL AND COMMUNITY CONTEXT**									
**Race**:			6.123	not applicable			not applicable		
Non-Hispanic Whites	1.05 **	1.02 *							
Racial/ethnic minorities (ref) ^a^	(1.01, 1.10)	(1.01, 1.12)							
**Marital status**:			6.323			0.523	not applicable		
Married/Partnered	1.10 **	1.06 *		1.13 *	1.01				
Divorced/Widowed/Single (ref)	(1.05, 1.16)	(1.01, 1.23)		(1.03, 1.24)	(0.98, 1.20)				
**EDUCATION**									
Bachelor’s/graduate or more High school or less/College(ref)	1.27 * (1.22, 1.32)	1.33 * (1.11, 1.52)	7.623	1.18 ** (1.07, 1.28)	1.06(0.98,1.19)	0.423	1.18 ** (1.11, 1.26)	1.11 * (1.01, 1.21)	4.651
**ECONOMIC STABILITY**									
**Employment Status**: Employed Unemployed/Homemakers/ Retired benefits (ref)	1.14 ** (1.12, 1.7)	1.12 ** (1.06, 1.13)	4.325	1.13 ** (1.07, 1.19)	1.13 ** (1.06, 1.19)	5.673	1.11 ** (1.06, 1.15)	1.09 ** (1.03, 1.14)	4.965
**Household Income/year**:			5.621			4.652			0.014
Equal to more than $35,000	1.11 **	1.08 **		1.10 **	1.07 *		1.07 **	0.96	
Less than $35,000 (ref)	(1.08, 1.14)	(1.05, 1.32)		(1.05, 1.16)	(1.01, 1.12)		(1.03, 1.10)	(0.92, 1.01)	
**Homeownership**:			0.162			0.123	not applicable.		
Own	1.25 **	1.11		1.37 **	1.11				
Rent (ref)	(1.12, 1.40)	(0.91, 1.25)		(1.11, 1.69)	(0.90, 1.36)				
**HEALTHCARE ACCESS**									
**Time since the last checkup with PCP**: Within the past year. More than one year ago/never (ref)	1.27 * (1.03, 1.56)	1.41 ** (1.13, 1.77)	8.753	1.38 * (1.02, 1.42)	1.46 * (1.01, 2.11)	6.451	1.41 * (1.02, 1.92)	1.49 * (1.07, 2.08)	8.912
**HEALTH RISK BEHAVIORS**									
**At least one alcoholic drink in the past 30 days**: No Yes (ref)	1.21 ** (1.21, 1.43)	1.39 ** (1.22, 1.56)	8.412	0.98 (0.6, 1.10)	1.21 (0.93, 1.56)	0.123	1.32 ** (1.09, 1.59)	1.21 (0.99, 1.48)	0.199
**Heavy drinking/week**:			4.641	not applicable			not applicable		
No	1.05	1.16 **							
Yes (ref)	(0.99, 1.13)	(1.02,1.26)							
**Smoking Status**:			7.312			7.521			0.312
Former/Never	1.25 **	1.21 **		1.28 **	1.21 **		1.12 *	1.02	
Current (ref)	(1.29, 1.30)	(1.14, 1.27)		(1.19, 1.36)	(1.01, 1.31)		(1.02, 1.20)	(0.91, 1.12)	
**Physical Activity:**			11.754			9.856			10.523
Yes	2.34 **	1.98 **		1.93 **	1.74 **		2.15 **	1.94 **	
No (ref)	(2.01, 2.73)	(1.71, 2.32)		(1.45, 2.51)	(1.33, 2.28)		(1.73, 2.71)	(1.55, 2.43)	
**Subset of the main dataset (2017, 2019, and 2021 with diet variables)**. **Weighted Study *N* = 123,261, *unweighted n = 835***									
	**Better General HRQoL** **(63%, Weighted *n* = 75,717)**			**Better Mental HRQoL** **(87%, Weighted *n* = 104,880)**			**Better Physical HRQoL** **(76%, Weighted *n* = 90,251)**		
**Diet (Vegetables):**			0.589			0.121			0.161
One or more times per day.	1.11	0.91		1.11	1.32		0.96	0.99	
Less than one time per day (ref)	(0.90, 1.23)	(0.78, 1.06)		(0.90, 1.26)	(0.91, 1.53)		(0.87, 1.06)	(0.85, 1.11)	

Note. * *p* < 0.05, ** *p* < 0.01. ^a^ Racial/ethnic minorities include Hispanic, Non-Hispanic Blacks, and Others; other GI Cancers include liver, esophageal, pancreatic, and stomach cancers. ^b^ R-squared Goodness of fit. We adjusted for survey years, age, types of GI cancers, and comorbidities in unadjusted and adjusted models as covariates. PCP, Primary Care Provider.

## Data Availability

Behavioral Risk Factor Surveillance System (BRFSS) survey data and the questionnaires are publicly available to researchers.
